# Description of *Plasmodium falciparum* infections in central Gabon demonstrating high parasite densities among symptomatic adolescents and adults

**DOI:** 10.1186/s12936-019-3002-9

**Published:** 2019-11-21

**Authors:** Rella Zoleko Manego, Erik Koehne, Andrea Kreidenweiss, Brice Nzigou Mombo, Bayode Romeo Adegbite, Lia Betty Dimessa Mbadinga, Malik Akinosho, Julian Matthewman, Ayola Akim Adegnika, Michael Ramharter, Ghyslain Mombo-Ngoma

**Affiliations:** 1 Centre de Recherches Médicales de Lambaréné, B.P. 242, Lambaréné, Gabon; 2 Institut für Tropenmedizin, Universität Tübingen, und Deutsches Zentrum für Infektionsforschung, Wilhelmstrasse 27, 72074 Tübingen, Germany; 3 Department of Tropical Medicine, Bernhard Nocht Institute for Tropical Medicine & I Dep. of Medicine, University Medical Center Hamburg-Eppendorf, Bernhard-Nocht-Straße 74, 20359 Hamburg, Germany

**Keywords:** Malaria, Parasitaemia, Adolescents, Adults, Gabon

## Abstract

**Background:**

Malaria remains a public health issue, particularly in sub-Saharan Africa with special features of seriousness in young children and pregnant women. Adolescents and adults are reported to have acquired a semi-immune status and, therefore, present with low parasitaemia. Children are understood to present with a much higher parasitaemia and severe malaria. It is a concern that effective malaria control programmes targeting young children may lead to a delay in the acquisition of acquired immunity and, therefore, causing a shift in the epidemiology of malaria. Prevalence and parasitaemia were explored in adolescents and adults with *Plasmodium falciparum* infections compared to young children in the area of Lambaréné, Gabon as an indicator for semi-immunity.

**Methods:**

A cross-sectional study was conducted at the Centre de Recherches Médicales de Lambaréné (CERMEL) during a 6-month period in 2018. Symptomatic patients, of all ages were screened for malaria at health facilities in Lambaréné and Fougamou and their respective surrounding villages in the central region of Gabon. *Plasmodium falciparum* infections were determined either by rapid diagnostic test (RDT) or by microscopy. Descriptive analysis of data on parasite densities, anaemia, and fever are presented.

**Results:**

1589 individuals screened were included in this analysis, including 731 (46%) adolescents and adults. Out of 1377 assessed, the proportion of *P. falciparum* positive RDTs was high among adolescents (68%) and adults (44%), compared to young children (55%) and school children (72%). Out of 274 participants assessed for malaria by microscopy, 45 (16%) had a parasite count above 10,000/µl of which 9 (20%) were adults.

**Conclusion:**

This study shows a high rate of *P. falciparum* infections in adolescents and adults associated with high-level parasitaemia similar to that of young children. Adolescents and adults seem to be an at-risk population, suggesting that malaria programmes should consider adolescents and adults during the implementation of malaria prevention and case management programmes with continuous care, since they also act as reservoirs for *P. falciparum*.

## Background

Malaria remains a leading cause of morbidity and mortality in sub-Saharan Africa despite the considerable progress made since the year 2000 [1–3]. The World Health Organization (WHO) estimated 219 million malaria cases worldwide and 435,000 malaria deaths in 2017 [4]. Some groups are considered at higher risk of becoming severely ill from *Plasmodium falciparum* infections in endemic regions, including pregnant women, infants, children under 5 years of age, and the non-immune migrants, as well as travellers.

There is evidence of age-dependent protection against severe malaria acquired by residents of high endemic malaria areas [5, 6]. Partial immunity is acquired gradually after repeated *P. falciparum* infections and is sequentially reflected first by a reduction of severe forms of malaria, then by a reduction in parasite density, but adults still can become infected and often remain asymptomatic [7, 8]. Age-dependent acquired immunity may be explained by the cumulative exposure to the large repertoire of *P. falciparum* antigen variants and also the fact that the immune mechanisms change with age [6, 8–10]. Consequently, adults continuously living in malaria endemic areas generally present with less severe symptoms and reduced parasite loads when infected by the parasite [11].

There is little known about the epidemiology of malaria and parasite level in the adult population in high transmission regions in comparison to what has been published about children, especially in Gabon. The prevalence of parasitaemia in the adult population remains an important issue because they act as a community reservoir for ongoing *Plasmodium* transmission [12]. There is concern that with control interventions targeting children under 5 years, the acquisition of immunity may be compromised, and the burden of malaria may shift to older children and adults [5]. In Gabon, the shift of the increased risk of malaria to children older than 5 years was reported in 2013 and hospital surveys have shown that half of adults with fever in urban areas had malaria [13, 14]. Studies characterizing malaria in adults from rural areas in Gabon are scarce. Results from a recent clinical trial from 2014 to 2016 in Lambaréné and Fougamou suggests that adults and adolescents above the age of 14 years experienced fewer symptoms with lower parasite densities than children [15]. This report aims to characterize the epidemiology of *P. falciparum* infections and its parasite density in the adolescent and adult population living in a rural endemic area of Gabon.

## Methods

### Study site and population

This prospective, cross-sectional analysis was done between January and June 2018. The data was retrieved from malaria diagnostic activities carried out on all patients seeking for health care at the Centre de Recherches Médicales de Lambaréné (CERMEL) in Gabon. Study participants came from Lambaréné, Fougamou, and surrounding villages, in the central region of Gabon, within the equatorial rainforest and highly endemic for *P. falciparum* malaria, which is perennial with little seasonal variation [16, 17]. Local strains of *P. falciparum* show high levels of resistance against chloroquine and sulfadoxine-pyrimethamine [18, 19].

Patients of all ages were routinely screened for *P. falciparum* infections and those positive by either rapid diagnostic test (RDT) or microscopy were invited through an informed consent process to participate in the respective clinical trials. All studies excluded pregnant and lactating women as well as those not consenting. This report included all individuals with a positive RDT or microscopy who consented to one of the clinical trials.

### Demographic and clinical characteristics

Demographic data including reported age, date of birth, gender, and place of residence were collected after an informed consent was obtained from the participants or their parents, or legal representatives in case of minors. Axillary temperature and haemoglobin levels were systematically recorded.

For this prospective analysis, participants were grouped into the following age categories: young children: ≤ 5 years of age, school children: 6 to 12 years of age, adolescents: 13 to 18 years of age, and adults: > 18 years of age.

### Laboratory assessments

Haemoglobin levels were assessed either with a Hemocue device (https://www.hemocue.com) or with an automated haematology analyzer (ABX Pentra 60, Horiba Medicals). Here, anaemia was defined as haemoglobin level < 10 g/dl, irrespective of the age and gender [20]. Fever was defined as axillary body temperature > 37.5 °C.

The malaria diagnostics used were both RDTs and microscopy. For the RDT, the Paracheck Pf^®^ Malaria Test (Orchid Biomedical Systems, India) was used. A positive test was confirmed with a second RDT of the same brand read by at least two different health care professionals. Thick blood smear microscopy slides were prepared according to the WHO method or the Lambaréné method as described elsewhere and stained with a 20% Giemsa solution for 20 min [21]. The result is the arithmetic mean of the results from two independent microscopists.

Asexual *P. falciparum* densities determined by microscopy were categorized into four groups, 0 parasites or “negative,” < 1000 parasites per µl or “low density,” 1000–9999 parasites per µl or “moderate density,” and ≥ 10,000 parasites per µl or “high density” [22, 23].

### Statistical analysis

Data recorded on screening logs were entered in an Excel table, and then imported for analysis using Stata version 13.1 (StataCorp, College Station, TX). The data is presented in tables with numbers and proportions for qualitative data while quantitative data like parasite density is presented as median and interquartile range (IQR). No hypothesis was tested, as this was a descriptive analysis.

## Results

**Fig. 1:**
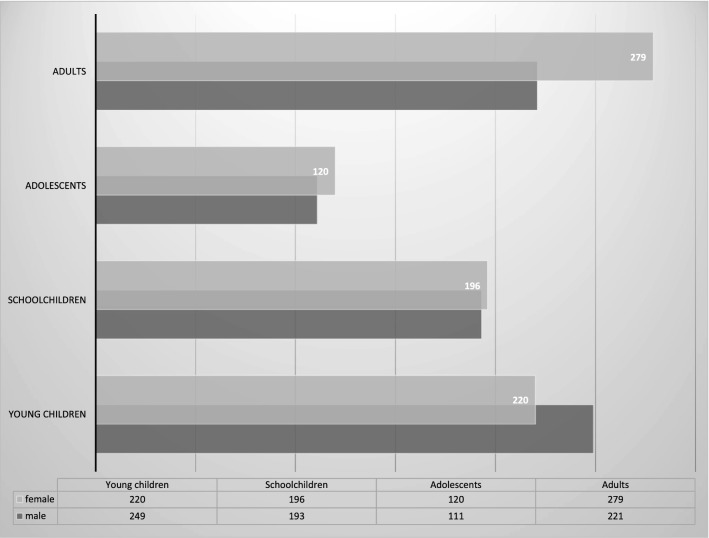
Demographic characteristics of participants by age groups

Of the subjects screened for malaria from January 9 to June 30, 2018, data was available for 1589 subjects included in this analysis as shown in Fig. 1. Of the 1589 subjects, 30% were young children and 24% were school children while the remaining subjects were adolescents (15%) and adults (31%), with median age (interquartile range) of 3 (2–5) years, 9 (7–11) years, 15 (14–17) years, and 33 (24–48) years, respectively. The gender was equally distributed within all age groups.

A total of 1377 subjects were screened for malaria by RDT and 792 (58%) were positive for *P. falciparum* (Table 1). Higher prevalence of positive RDTs were observed in the school age children (72%) and adolescents (68%) while the rate of positive RDTs was below the average in young children (55%) and adults (44%). Microscopy screening of malaria was systematically performed during January 2018 for 274 subjects with 159 (58%) positive for *P. falciparum* infection and the proportions of positives within the different age groups were similar to the RDTs, as shown in Table 1. Parasite densities of the 274-microscopy results are shown in Table 2. Among the highest parasite densities > 10,000, the school age children were the most frequent (47%) followed by adults (20%).

**Table 1:** Prevalence of *Plasmodium falciparum* infections according to age group and diagnostic test

	RDT		Microscopy	
	Positive, N (%)	Total	Positive, N (%)	Total
Young children	230 (55)	421	22 (47)	47
School children	231 (72)	321	49 (69)	71
Adolescents	139 (68)	203	34 (60)	57
Adults	192 (44)	432	54 (55)	99
Total	792 (58)	1377	159 (58)	274

RDT, rapid diagnostic test; N, number; %, percentage

**Table 2:** Parasite densities and distribution in age groups

Parasite density (pf/µl)	0	< 1000	1000–9999	≥ 10,000	Total
Median parasite density (Pf/µl) [IQR]	0	223 [108–572]	3483 [2295–5492]	33,393 [17,190–44,111]	3450 [696–12,600]
N	115	50	64	45	274
Young children, n (%)	25 (22)	6 (12)	9 (14)	7 (15)	47 (17)
School children, n (%)	22 (19)	9 (18)	19 (30)	21 (47)	71 (26)
Adolescents, n (%)	23 (20)	15 (30)	11 (17)	8 (18)	57 (21)
Adults, n (%)	45 (39)	20 (40)	25 (39)	9 (20)	99 (36)

Pf, *plasmodium falciparum*; IQR, interquartile range; N, total number; n (%), number (percentage)

During that same month of January, data on haemoglobin level and body temperature were available for 256 and 264 subjects, respectively. The overall median (IQR) haemoglobin level was 10.7 (9.3–11.9) g/dl, while for young children, school children, adolescents and adults the median (IQR) haemoglobin levels were 9.6 (7.9–10.8) g/dl, 10.7 (9.7–11.8) g/dl, 11.3 (10.2–12.4) g/dl, and 11.4 (10.0–13.2) g/dl, respectively. The young children age group was the most affected while all the other groups had proportions of anaemic less than 30% as shown in Table 3. Overall, fever was observed in 30% of the examined subjects with the children in general more febrile. However, more than 20% of the adolescents and adults presented with fever (Table 3).

**Table 3:** Proportion of anaemia and fever in the subset assessed in January 2018

	Anaemia		Fever	
	Yes, N (%)	Total	Yes, N (%)	Total
Young children	43 (61)	70	24 (34)	70
School children	26 (29)	91	33 (36)	91
Adolescents	8 (19)	42	11 (24)	46
Adults	13 (24)	53	12 (21)	57
Total	90 (35)	256	80 (30)	264

N (%), number and percentage

## Discussion

Children under five years and pregnant women in high malaria endemic regions are the most vulnerable to malaria and studies mainly focus on these populations. *Plasmodium falciparum* infection in adults is reported as being of low parasitaemia due to age-dependent acquired immunity [6, 24]. Rarely do adults become severely ill to malaria in hyperendemic malaria areas [5]. This study conducted in Lambaréné and Fougamou showed an overall *P. falciparum* infection rate of 58%, with school children and adolescents bearing the highest proportions of infections, with adults as much infected as young children. The findings are similar to the prevalence found in adults in Libreville, the capital of Gabon, in 2012 [13]. This is different from other findings of 21% prevalence in asymptomatic populations in a study conducted in rural areas of Gabon in 2014 [25]. The observed difference can be explained by the difference of the population as our population was made of patients seeking for care which is different of asymptomatic adults certainly screened for malaria in their households. In Kenya and Mozambique, the prevalence of *P. falciparum* infections in adults was reported to be approximately 28% and 14%, respectively [12, 26]. These results correspond with the results of Bouyou-Akotet et al. supporting the hypothesis that there may be a change in the epidemiological profile of *P. falciparum* infections in adolescents and adults. This high prevalence of *P. falciparum* reported by other studies could also be explained by a decrease in malaria control strategies, such as a reduction of the free distribution of insecticide-treated bed nets and the lack of re-impregnation of used bed nets [3, 27].

The highest rate of *P. falciparum* infections was found in school children and adolescents in this study. This finding suggests a shift of malaria risk from very young children to school-age children, which is in line with the results reported by Mawili-Mboumba et al. who stated that children above 5 years of age would now become an at-risk population for malaria [13]. Substantial declines in malaria transmission, morbidity, and mortality in numerous countries where new malaria control strategies have been implemented were reported by several studies [28, 29]. These declines are more important in children under 5 years of age, the authors also suggested malaria control strategies to be extended to older children, and adults to reduce the prevalence since these groups may act as reservoirs for *P. falciparum* transmission.

Proportions of people with high parasite densities in young children, adolescents, and adults were similar with 15%, 18%, and 20%, respectively, having a high parasitaemia of ≥ 10,000 *P. falciparum*/µl while the proportion was much higher for school children (47%). Studies suggest that semi-immunity to *P. falciparum* develops with age based on cumulative exposure to the parasites, therefore, parasite densities are much lower in adults than in children [5, 6]. This data suggests no major difference in parasitaemia between young children, adolescents, and adults, which may further highlight the possibility of a shift in *P. falciparum* infections in the general population in Gabon.

Approximately one-third of the participants had anaemia, which may be related to the presence of a *P. falciparum* infection. The majority of anaemic individuals had haemoglobin values between 6 and 10 g/dl with only a few participants having severe anaemia, similar to reports in Cameroon and Thailand [30, 31]. No other infections were found in the participants, but nutritional deficiencies and malaria are recognized as the main causes of anaemia in African children [32]. Other factors contributing to anaemia include infections such as hookworm, helminthiasis, and HIV-AIDS. Soil-transmitted infections were not searched in this study. This study contributes to the description of *P. falciparum* infections in adults in a high transmission area. One limitation to this study is the absence of a full parasitological diagnosis for all participants as the finding related to parasite density may be specific to the only month of January and not be generalized to the other periods of the year. Nonetheless, it was observed that the prevalence of malaria during the 6-month period assessed by RDT was similar to the 1-month period assessed by microscopy. Further studies are necessary to more extensively characterize *P. falciparum* infections and prevalence in adolescents and adults in Gabon as adolescents and adults may be at greater risk of malaria with the control programmes oriented at earlier life periods.

## Conclusions

Global efforts for control of malaria have been targeting infants and children below 5 years and pregnant women because they are described as the populations’ the most affected by the malaria burden in terms of morbidity and mortality. The children once targeted by control programmes mature to adolescence and then adulthood becoming vulnerable to malaria. Indeed, the reduced exposure to *P. falciparum* due to the effective control programmes during their younger ages may have delayed their acquisition of the acquired immunity. Therefore, there is a need for malaria control programmes to assure a continuum of care that includes adolescents and adults of both sexes.

## Data Availability

The datasets used and/or analysed during the current study are available from the corresponding author on reasonable request.

## Abbreviations

CERMEL: Centre de Recherches Médicales de Lambaréné
RDT: rapid diagnostic test
WHO: World Health Organization
IQR: interquartile range
